# Regulating the properties of XQ-2d for targeted delivery of therapeutic agents to pancreatic cancers

**DOI:** 10.1093/nsr/nwad113

**Published:** 2023-04-25

**Authors:** Qiuxia Yang, Yongbo Peng, Zhengyu Deng, Dailiang Zhang, Cheng-Yu Long, Guo-Rong Zhang, Juan Li, Xue-Qiang Wang, Weihong Tan

**Affiliations:** Zhejiang Cancer Hospital, Hangzhou Institute of Medicine (HIM), Chinese Academy of Sciences, Hangzhou 310022, China; Molecular Science and Biomedicine Laboratory (MBL), State Key Laboratory of Chemo/Biosensing and Chemometrics, College of Chemistry and Chemical Engineering, College of Biology, Aptamer Engineering Center of Hunan Province, Hunan University, Changsha 410082, China; Molecular Science and Biomedicine Laboratory (MBL), State Key Laboratory of Chemo/Biosensing and Chemometrics, College of Chemistry and Chemical Engineering, College of Biology, Aptamer Engineering Center of Hunan Province, Hunan University, Changsha 410082, China; Zhejiang Cancer Hospital, Hangzhou Institute of Medicine (HIM), Chinese Academy of Sciences, Hangzhou 310022, China; Molecular Science and Biomedicine Laboratory (MBL), State Key Laboratory of Chemo/Biosensing and Chemometrics, College of Chemistry and Chemical Engineering, College of Biology, Aptamer Engineering Center of Hunan Province, Hunan University, Changsha 410082, China; Molecular Science and Biomedicine Laboratory (MBL), State Key Laboratory of Chemo/Biosensing and Chemometrics, College of Chemistry and Chemical Engineering, College of Biology, Aptamer Engineering Center of Hunan Province, Hunan University, Changsha 410082, China; Zhejiang Cancer Hospital, Hangzhou Institute of Medicine (HIM), Chinese Academy of Sciences, Hangzhou 310022, China; Zhejiang Cancer Hospital, Hangzhou Institute of Medicine (HIM), Chinese Academy of Sciences, Hangzhou 310022, China; Molecular Science and Biomedicine Laboratory (MBL), State Key Laboratory of Chemo/Biosensing and Chemometrics, College of Chemistry and Chemical Engineering, College of Biology, Aptamer Engineering Center of Hunan Province, Hunan University, Changsha 410082, China; Zhejiang Cancer Hospital, Hangzhou Institute of Medicine (HIM), Chinese Academy of Sciences, Hangzhou 310022, China; Molecular Science and Biomedicine Laboratory (MBL), State Key Laboratory of Chemo/Biosensing and Chemometrics, College of Chemistry and Chemical Engineering, College of Biology, Aptamer Engineering Center of Hunan Province, Hunan University, Changsha 410082, China; Institute of Molecular Medicine (IMM), Renji Hospital, School of Medicine, College of Chemistry and Chemical Engineering, Shanghai Jiao Tong University, Shanghai 200127, China

**Keywords:** phosphorothiolation, MMC-functionalized aptamer, targeted pancreatic cancer therapy

## Abstract

Enhanced recognition ability, cell uptake capacity, and biostability are characteristics attributed to aptamer-based targeted anticancer agents, and are possibly associated with increased accumulation at the tumor site, improved therapeutic efficacy and reduced negative side effects. Herein, a phosphorothioate backbone modification strategy was applied to regulate the biomedical properties of pancreatic cancer cell–targeting aptamer for efficient *in vivo* drug delivery. Specifically, the CD71- targeting aptamer XQ-2d was modified into a fully thio-substituted aptamer S-XQ-2d, improving the plasma stability of S-XQ-2d and mitomycin C (MMC)-functionalized S-XQ-2d (MFSX), thus considerably prolonging their half-life in mice. Moreover, the binding and uptake capacities of S-XQ-2d were significantly enhanced. MFSX showed the same level of cytotoxicity as that of MMC against targeted cancer cells, but lower toxicity to non-targeted cells, highlighting its specificity and biosafety. Brief mechanistic studies demonstrated that XQ-2d and S-XQ-2d had different interaction modes and internalization pathways with the targeted cells.

## Teaser

A full phosphorothioate backbone modification strategy was developed to regulate the biomedical properties of pancreatic cancer cell-targeting aptamer for efficient *in vivo* drug delivery.

## INTRODUCTION

Pancreatic cancer is the most lethal solid tumor owing to its difficulty to diagnose and treat, leading to approximately 227 000 deaths annually worldwide [1]. Pancreatic ductal cancer, as the main type of pancreatic cancer, accounts for most cases (>90%), and the overall survival time of patients with this type of cancer is approximately 6 months from diagnosis. Pancreatic cancer is prone to high local invasion and distant metastases, and the treatment outcomes usually depend on the severity of the disease at diagnosis. Adjuvant therapy combined with surgical resection is the standard of care for patients diagnosed with an early-stage disease. However, since most patients present with advanced-stage disease, they usually respond poorly to surgery [2,3]. In this context, extensive efforts have been undertaken to develop innovative therapeutic strategies to improve pancreatic cancer treatment outcomes, including anti-signal transduction pathway regulation, cell therapy and gene transcription-level regulation therapy [4,5]. These treatments involve the use of molecular biomarkers to detect cancer, determine prognosis and monitor disease progression or therapeutic response.

As part of our research interest in aptamer-mediated targeted cancer therapy, we recently described a strategy to enhance synergistic anticancer effects *via* conjugation of XQ-2d, a CD71-targeting aptamer, and mitomycin C (MMC), a DNA alkylation reagent, with a reductant-responsive disulfide linker [6]. Although this targeted drug delivery technique allows for efficient inhibition of pancreatic cancer cell proliferation, rapid nuclease-mediated degradation of MMC-functionalized XQ-2d (MFX) was observed in the culture medium, raising concerns about elevated off-target risk. Modification strategies, including loading on nanoparticles [7], adjusting DNA chirality [8,9], and using artificial nucleosides [10], have been developed to enhance the stability of nucleic acids against nuclease. Moreover, phosphorothioate (PS) backbone modification (PSBM) is a common strategy for overcoming the instability of nucleic acids [11,12], and possesses unique advantages, for instance: thiolated nucleic acids can be conveniently synthesized using an automated DNA synthesizer in a site-controllable manner. Furthermore, owing to differences in electronegativity and polarity between sulfur and oxygen atoms, PS nucleic acids show an obvious advantage in terms of protein binding, cellular uptake, subcellular localization and distribution kinetics [13–15]. For example, antisense oligonucleotides (ASOs) modified with PS linkage, a representative model, show higher hydrophobicity, nuclease resistance, and affinity to proteins than ASOs with phosphodiester linkages [16–18]. Moreover, benefiting from the excellent biological characteristics of PS linkages, many PS nucleic acids are currently at various stages of clinical development, and some have even been approved by the FDA [19,20].

**Scheme 1.**
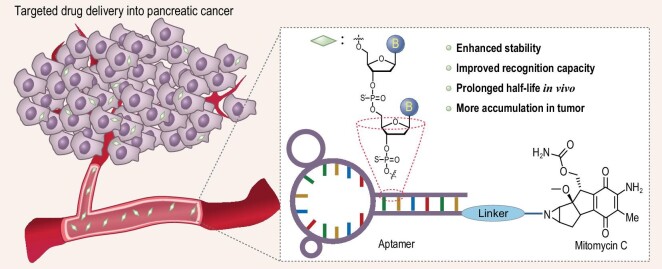
A brief illustration of XQ-2d PS-modification for drug delivery in pancreatic cancer.

In this work, we studied the influence of the full thio-substitution of the XQ-2d aptamer and its conjugation with the chemotherapy drug MMC (Scheme 1). Our results revealed that the full PS linkage modification enhanced the stability, binding ability, and cell endocytosis of the original aptamer, and these findings differ from those of terminal modification [21]. The strengthened interaction between S-XQ-2d and cells, and the change in internalization pathways accelerated S-XQ-2d endocytosis into its target cells. Importantly, MMC-functionalized S-XQ-2d (MFSX) showed the same level of cytotoxicity as the small-molecule drug MMC against targeted cancer cells, but considerably lower toxicity to non-target cells. Thus, these results suggest that full PS modification could provide a new method of modulating the activity of aptamers and aptamer-based diagnosis and treatment agents, as well as promoting their clinical transformation.

## RESULTS AND DISCUSSIONS

### Synthesis and characterization of MFX, MFSX and MFC

The aptamer XQ-2d, previously selected through CELL-SELEX by our group, was chosen as our research model [21]. This aptamer binds strongly and specifically to the protein receptor CD71, which is highly expressed on the membrane of cancer cells, such as PL45 cells [22]. Encouraged by these findings, we previously developed XQ-2d-MMC conjugates to significantly enhance the cytotoxicity and specificity of the non-target drug MMC [6]. However, the low resistance to metabolic degradation largely restricts the *in vivo* therapeutic applications of these conjugates. As previously reported, PS modification increases the stability of nucleic acids. Thus, we reasoned that this strategy might address the issue discovered with the XQ-2d-MMC conjugates, as noted above. To test this idea, we first synthesized S-XQ-2d using a PS modification strategy (Scheme S1), which allowed us to determine any variation in metabolic stability, targeting or internalization abilities. MMC was conjugated to S-XQ-2d via a 4-nitrophenyl 2-(2-pyridyldithio) ethyl carbonate linker. This aptamer-based drug conjugate can be disintegrated by intracellular reductant reagents to release MMC. The conjugate was purified by high-performance liquid chromatography and characterized with mass spectrometry (Figure S1). An MMC-functionalized control sequence (MFC), which had no binding capacity to PL45 cells, was synthesized as a negative control [6].

### Cell targeting ability of S-XQ-2d and MFSX

After PS modification, S-XQ-2d was identified to bind its target CD71 protein with a KD value of 2.9 nM, which is approximately 13-fold stronger than XQ-2d of 39.1 nM (Figures S2, S3 and Table S1), indicating that the PS modification could tremendously increase the interaction between the aptamer and the target protein. Next, we studied whether PS modification affected the binding ability against cells. Cy5-XQ-2d, Cy5-S-XQ-2d, Cy5-MFSX, and Cy5-MFC were separately measured for their binding ability to PL45 and MCF-7 cells (control cell line with low CD71 expression) by flow cytometry. Interestingly, the S-XQ-2d and MFSX groups exhibited enhanced fluorescence intensity compared with the other PL45 cell groups (Figure S4). We also observed a slight increase in the binding ability of S-XQ-2d and MFSX to MCF-7 cells, possibly due to nonspecific interactions with cell membrane proteins [12].

**Figure 1.**
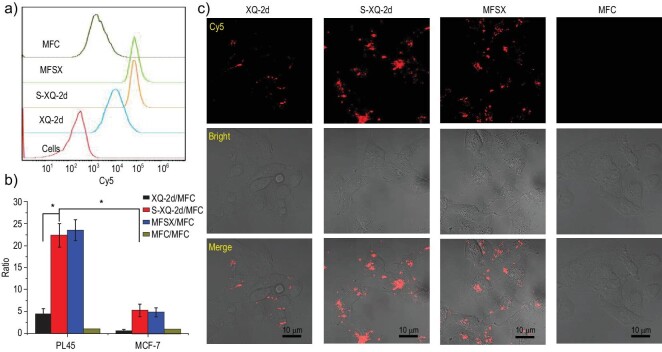
Assays to determine enhanced selective internalization ability. (a) Cell uptake efficiency of Cy5-XQ-2d, Cy5-S-XQ-2d, Cy5-MFSX and Cy5-MFC by PL45 cells (250 nM, incubated for 2 h at 37°C), measured by flow cytometry. (b) The ratio of Geometric Mean Fluorescence Intensity to Cy5-MFC for Cy5-XQ-2d, Cy5-S-XQ-2d, or Cy5-MFSX uptake by PL45 cell or MCF-7 cells. (c) Endocytosis by PL45 cells characterized by confocal images. The cells were treated with 250 nM of Cy5-XQ-2d, Cy5-S-XQ-2d, Cy5-MFSX and Cy5-MFC, respectively for 2 h at 37°C. Scale bar = 10 μm.

To investigate how PS modification influences the selective internalization ability of aptamers, we explored the cellular uptake of thiolated XQ-2d and its conjugates using flow cytometry and confocal imaging. First, we found that the uptake of S-XQ-2d, or MFSX, by PL45 cells was more efficient than that of XQ-2d and the control MFC, as presented in Fig. 1 (a and b). Moreover, the endocytosis ratio of S-XQ-2d and MFSX to XQ-2d in PL45 cells was much higher than that in MCF-7 cells (Fig. 1b), implying that PS modification could considerably improve the selectivity of the aptamer [11,12]. To further confirm this conclusion, confocal imaging was used to record the internalization events of XQ-2d, S-XQ-2d and MFSX. As pictured in Fig. 1c, the cellular uptake of S-XQ-2d and MFSX was much higher than that of XQ-2d, as revealed by the brighter fluorescence intensity.

**Figure 2.**
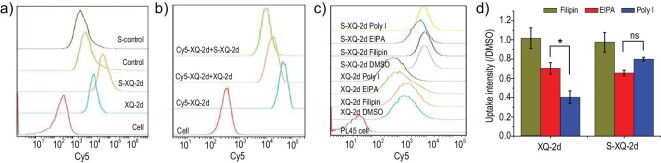
Enhanced binding ability and internalization pathway determination assays. (a) Binding ability of Cy5-XQ-2d, Cy5-S-XQ-2d, Cy5-control and Cy5-S-control to PL45 cells at a concentration of 250 nM at 4°C for 50 min. (b) Binding competition of S-XQ-2d and XQ-2d. Cy5-XQ-2d (500 nM) was preincubated with PL45 cells for 30 min at 4°C. After washing with washing buffer 3 times, S-XQ-2d or XQ-2d (1 μM) was separately incubated with pretreated cells to compete with Cy5-XQ-2d for another 30 min at 4°C. (c) Endocytosis inhibition assays. Filipin (4 μM), EIPA (100 μM), Poly I (40 μg/mL) or DMSO (4 μL) was incubated with PL45 cells for 45 min at 37°C, respectively. Then, 250 nM Cy5-XQ-2d or Cy5-S-XQ-2d, were added and incubated for another 2 h at 37°C. The uptake amount was measured by flow cytometry and DMSO was used as a control sample. (d) Relative uptake intensity compared to the DMSO control group.

To verify this result further, we synthesized a Cy5-labeled fully phosphorothiolated control sequence (Cy5-S-control) and tested its binding ability (Figs 2a and S5). The fluorescence intensity of the Cy5-S-control strand did not exceed that of Cy5-control for PL45 or MCF-7 cells, indicating that phosphorothiolation could not promote the binding of the control sequence. In addition, we conducted competitive assays in which moderate amounts of Cy5-XQ-2d (500 nM) were preincubated with PL45 cells, after which adequate amounts of S-XQ-2d or XQ-2d (1 μM) were separately incubated with pretreated cells to compete with Cy5-XQ-2d. The fluorescence intensity of the remaining Cy5-XQ-2d was measured using flow cytometry (Fig. 2b). The sample treated with S-XQ-2d had a weaker intensity than that treated with XQ-2d, owing to more Cy5-XQ-2d separated from cells under competition from S-XQ-2d. These results demonstrated that the enhanced cellular uptake ability, after complete thiolation of the aptamer, mainly accounted for the strengthened CD71-aptamer interaction and the effect only occurred for the aptamer, but not for the control strand.

Apart from the interaction with receptors, cellular trafficking can also determine the endocytosis ability of foreign substrates, such as nucleic acids [23–25]. To investigate the difference between the uptake pathways of XQ-2d and S-XQ-2d, we examined the effect of different endocytosis inhibitors, including the uptake of XQ-2d and S-XQ-2d by PL45 cells. We first incubated filipin (inhibitor of caveola), EIPA (inhibitor of macropinocytosis) and Poly I (inhibitor of scavenger receptor, SR) with PL45 cells for 45 min at 37°C using 4 μL DMSO as a control treatment [26]. Cy5-XQ-2d or Cy5-S-XQ-2d (250 nM) was then added and incubated for another 2 h at 37°C, and aptamer uptake was measured by flow cytometry. As shown in Fig. 2 (c and d), the inhibition ability of Poly I was the strongest for XQ-2d, indicating that XQ-2d was mainly taken up by SR, and partly by macropinocytosis. The cellular internalization of S-XQ-2d was closely influenced by EIPA and Poly I, implying that the endocytosis of S-XQ-2d was SR- and micropinocytosis-dependent. These two strands were taken up in a caveola-independent manner, as no effect on the fluorescence intensity of filipin was observed. The results revealed that the difference in cellular uptake and trafficking might also account for the enhanced uptake of S-XQ-2d by the targeted PL45 cells.

### Cell cytotoxicity of MFSX

**Figure 3.**
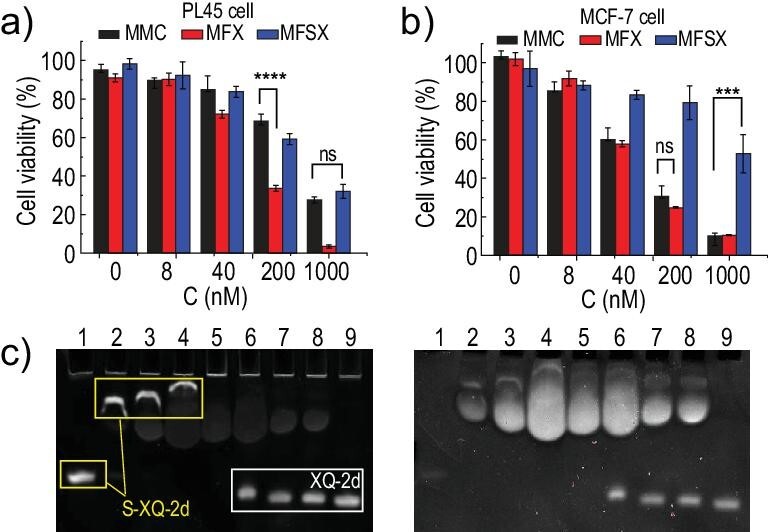
Cell cytotoxicity of MMC, MFX and MFSX. Cell viability of (a) PL45 cells and (b) MCF-7 cells after incubation with different concentrations of MFX or MFSX (1.6, 8, 40, 200 and 1000 nM) for 72 h at 37°C. (c) The interaction between XQ-2d or S-XQ-2d with BSA; 2 μM DNA strands were incubated with BSA water solution for 2 h at room temperature and then characterized by Native-PAGE. Lane 1: S-XQ-2d; lane 2: S-XQ-2d with 1% BSA; lane 3: S-XQ-2d with 2% BSA; lane 4: S-XQ-2d with 5% BSA; lane 5: 2% BSA; lane 6: XQ-2d with 5% BSA; lane 7: XQ-2d with 2% BSA; lane 8: XQ-2d with 1% BSA; lane 9: XQ-2d. The gels were stained with Super Gelred^TM^ (left) and Coomassie Brilliant Blue (right).

After determining the excellent binding and internalization capacities of S-XQ-2d, we conducted cell cytotoxicity experiments to assess its anticancer function. Unexpectedly, the IC_50_ value of MFSX was higher than that of MFX, but approximate to that of naked MMC against PL45 cells (MFSX: 328.6 nM; MFX: 95.02 nM; MMC: 382.2 nM) [6]. MFSX showed lower toxicity than MFX and MMC compared to control MCF-7 cells (MFSX: 951.0 nM; MFX: 63.84 nM; MMC: 71.38 nM), as shown in Fig. 3 (a and b). These observations suggested that fully thio-modified MFSX has the same level of cytotoxicity as MMC, but reduced side effects on non-target cells due to their enhanced specific recognition capability.

However, the lower toxicity of MFSX compared with that of MFX was undesirable. We speculated that the reduced cancer cell–killing ability resulted from the MFSX-protein interaction. Since phosphorothiolated nucleic acids exhibit nonspecific binding behavior with serum proteins, including human or bovine serum albumin (HSA or BSA) [27,28], we explored the binding behavior of S-XQ-2d and XQ-2d to BSA using gel electrophoresis to rationalize the reduced cytotoxicity of MFSX. We first incubated different amounts of BSA (0%, 1%, 2% and 5%) with 2 μM XQ-2d or S-XQ-2d for 2 h at 25°C, and then characterized their binding status by Native-PAGE. We found that S-XQ-2d was completely adsorbed when incubated with a 2% BSA solution. In contrast, XQ-2d was observed in solution under the same incubation conditions (Fig. 3c). This result indicated that S-XQ-2d had a stronger capacity to bind to BSA than to XQ-2d, which might explain the reduced anticancer effect of MFSX compared to that of MFX. Next, we explored the influence of BSA on MFSX cytotoxicity. We measured cell viability after the treatment of PL45 cells with 1 μM MFSX for 72 h in the presence of different amounts of BSA. As shown in Fig. S6, the cytotoxicity of MFSX with BSA was lower than that without BSA, demonstrating that the interaction between BSA and S-XQ-2d impaired the cell-killing ability of MFSX.

### Biological stability and *in vivo* distribution

**Figure 4.**
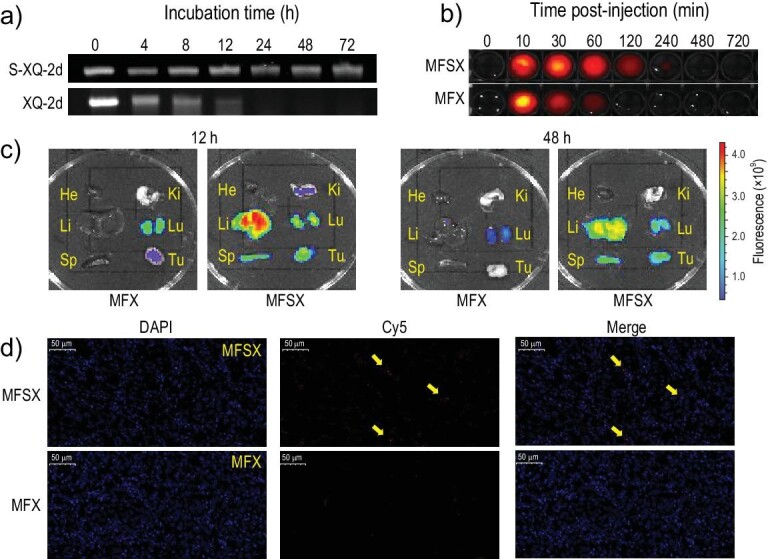
Stability and accumulation determination assays of aptamers and MFX. (a) Stability of S-XQ-2d (2 μM) or XQ-2d (2 μM) in DMEM culture medium containing 20% FBS at 37°C for 0, 4, 8, 12, 24, 48 and 72 h, as characterized by native polyacrylamide gel electrophoresis (Native-PAGE). The gels were stained with Super Gelred^TM^. (b) The fluorescence imaging of blood samples extracted from mice at different time points post-injection of 20 nmol Cy5-MFX or Cy5-MFSX. (c) Biodistribution of MFX and MFSX (Tu: tumor, He: heart, Li: liver, Sp: spleen, Lu: lung, Ki: kidney) 12 h and 48 h post-injection. (d) The fluorescence intensity of MFX and MFSX in the tumor at 48 h. The cell nucleus was stained with DAPI.

Reportedly, PS modification at either specific or full sites is helpful in restricting enzyme degradation and improving the metabolic stability of therapeutic ASOs and short-interfering RNAs [17]. To determine any change in the pharmacological properties of XQ-2d after phosphorothiolation, we separately mixed S-XQ-2d and XQ-2d with DMEM containing 20% FBS and incubated them at 37°C for different time periods. As demonstrated by the electrophoresis results (Fig. 4a), S-XQ-2d had better serum stability than XQ-2d. S-XQ-2d remained in the medium for more than 72 h, whereas XQ-2d was almost completely degraded after 12 h. To explore the potential of using S-XQ-2d for targeted cancer therapy, we first prepared Cy5-MFSX and Cy5-MFX to compare their *in vivo* stability. Prior to this, the standard curves of Cy5-MFSX and Cy5-MFX in rat blood were fitted by mixing a series of conjugates (0, 0.05, 0.1, 0.2, 0.4, 0.8 and 1 μM) with 100 μL of rat blood (Fig. S7). Conjugates (20 nmol) were then injected into rats *via* tail vein injection, and after 0, 10, 30, 60, 120, 240, 480 and 720 min, blood was drawn from the rat eyes to measure the fluorescence signal. As indicated in Fig. 4b, MFSX lasted much longer than MFX in the blood. The speculated half-life of MFSX was approximately 5-fold longer than that of MFX, and the plasma clearance rate (CL) was one-sixth (Table S2), demonstrating that S-XQ-2d possesses higher biostability than XQ-2d.

We also examined another *in vivo* application of MFSX: targeting and accumulation. *In vivo* imaging of Cy5-MFX and Cy5-MFSX indicated that more MFSX accumulated in tumor tissues than MFX. It is worth noting the near absence of fluorescence at the tumor site 4 h after Cy5-MFX injection, whereas signals were detected even at 48 h for Cy5-MFSX (Fig. S8). Imaging of other organs revealed that MFSX was primarily accumulated in the liver with a long residence time (Fig. 4c), which might be explained by the strong detoxification function of the liver. Importantly, the fluorescence intensity of MFSX in the tumor was much higher than that of MFX. In tumor tissue sections, the stronger Cy5-MFSX signal at 48 h indicated that MFSX was more likely to accumulate and penetrate deeper into tumor tissue and was more metabolically resistant compared to MFX, consistent with the longer half-life of S-XQ-2d (Fig. 4d). These results confirmed that S-XQ-2d had a higher resistance to enzyme digestion than XQ-2d, thus highlighting the potential of utilizing MFSX for pancreatic cancer treatment.

## CONCLUSIONS

The phosphorothioate modification of nucleic acids is widely used to optimize their physiological functions. We replaced all PO linkages with PS in the XQ-2d aptamer for the first time and conjugated them with an anticancer molecule to probe alterations in nuclease resistance, targeting ability and anticancer effects. The physical stability of S-XQ-2d and MFSX was improved. In addition, the targeted anticancer effect of MFSX was retained, and the side effects of binding to untargeted cells were reduced, advancing its therapeutic potency. Interestingly, the binding affinity and endocytosis of S-XQ-2d and MFSX were greatly augmented. The enhanced uptake ability may possibly be attributed to the stronger interaction between S-XQ-2d and the targeted cells and different internalization pathways. Our results revealed how PS linkage modifications could affect the physiological status of the original XQ-2d aptamer and regulate its therapeutic efficiency more easily and in a purposeful manner. We expect that this PS modification strategy will accelerate the clinical application of aptamers and aptamer-related diagnostic and treatment tools.

## MATERIALS AND METHODS

All reaction reagents were purchased from Energy Chemical. Mitomycin C was purchased from Sanlidobio Co., Ltd. (Nanjing) and stored at −20°C. DNA strands from Beijing Hippo Biotechnology Co., Ltd. and DNA strands were dissolved in ultrapure water (Milli-Q). All DNA-drug conjugates were purified by reversed-phase HPLC (ProStar, Varian, Walnut Creek, CA, USA) on a C-18 column using 0.1 M triethylamine acetate (TEAA Glen Research Corp.) and acetonitrile (Sigma Aldrich, St. Louis, MO, USA) as the eluent. CD71 protein was purchased from Sino Biological (11020-H07H). CCK-8 (Cell Counting Kit-8) was purchased from New Cell & Molecular Biotech. Super GelRed^TM^, 10 000 × in water was purchased from US Everbright^®^ Inc. Cell culture dishes or flasks were purchased from NEST Biotechnology.

## Supplementary Material

nwad113_Supplemental_File
