# Supplementary material for: Regulating the properties of XQ-2d for targeted delivery of therapeutic agents to pancreatic cancers
Source: Natl Sci Rev. 2023 Apr 25;10(8):nwad113. doi: 10.1093/nsr/nwad113 (PMC10508320; doi:10.1093/nsr/nwad113)

**Supplementary Materials**

**Regulating the Properties of XQ-2d for Targeted Therapeutic Agents Delivery to Pancreatic Cancer**

Qiuxia Yang,^a^ Yongbo Peng,^b^ Zhengyu Deng,^b^ Dailiang Zhang,^a^ Cheng-Yu Long,^b^ Guo-Rong Zhang,^b^ Juan Li,^a^ Xue-Qiang Wang,*^, a, b^ Weihong Tan*^, a, b, c^

^a^ Zhejiang Cancer Hospital, Hangzhou Institute of Medicine (HIM), Chinese Academy of Sciences, Hangzhou, 310022, China

^b.^Molecular Science and Biomedicine Laboratory (MBL), State Key Laboratory of Chemo/Biosensing and Chemometrics, College of Chemistry and Chemical Engineering, College of Biology, Aptamer Engineering Center of Hunan Province, Hunan University, Changsha, 410082, China

^c^ Institute of Molecular Medicine (IMM), Renji Hospital, School of Medicine, College of Chemistry and Chemical Engineering, Shanghai Jiao Tong University, Shanghai 200127, China

Corresponding author: [wangxq@hnu.edu.cn](mailto:wangxq@hnu.edu.cn); [tan@hnu.edu.cn](mailto:tan@hnu.edu.cn).

Supplementary Materials and Methods

**Synthesis and characterization of MFSX, MFX, and MFC**

We adopted NPEC linkers to conjugate DNA strands with MMC following a previously reported procedure. MFX and MFC were synthesized and characterized following a described work. For MFSX, XQ-2d strands with all P=S replacement in phosphate backbone and sulfo-C_6_ modification in the 5’-terminal in water were added into NPDEC-MMC in DMF at the ratio of DNA:MMC-linker=1:300, followed by shaking at 37°C overnight. The products were purified by HPLC and characterized by mass spectra.

**Cell culture**

The cell lines were all obtained from ATCC (American Type Culture Collection, Manassas, VA). To culture PL45 cells, Dulbecco’s modified Eagle’s medium (DMEM) containing 20% (v/v) fetal bovine serum (FBS) was used. MCF-7 cells were cultured in RPMI-1640 medium supplemented with 10% fetal bovine serum (FBS). They were both incubated at 37°C with 5% CO_2_. Short-term (1-2 min) trypsin treatment was adopted to dissociate cells from the culture flask or dish.

Binding ability of DNA strands by SPR

SPR experiments were performed on a Biacore8K instrument (GE Healthcare). CD71 was immobilized on a Series S CM5 chip in an immobilization buffer (pH 4.0) containing 10 mM CH_3_COONa at 25°C. For binary studies, the final surface density of CD71 was approximately 6500–7000 RU. All interaction experiments were performed in a running buffer (pH 7.4) containing 8.1 mM Na_2_HPO_4_, 137 mM NaCl, 2.68 mM KCl,1.47 mM KH_2_PO_4_, 0.05% Tween 20, and 4% DMSO at 25°C. Gradient concentrations of aptamers were injected at a flow rate of 30 μL/min. The association time is 180 s and the dissociation time is 180 s.

Binding ability of DNA strands and conjugates measured by flow cytometry

All DNA strands were labeled with Cy5 at the 3’-terminal to explore the target-binding ability of XQ-2d, S-XQ-2d, Control, S-control, MFX, MFSX, and MFC. PL45 cells and MCF-7 cells were dissociated from the culture dish with 1 mL of 0.2% EDTA and washed twice by washing buffer (DPBS containing 4.5 g/L glucose, 5 mM MgCl_2_, and 1 mg/mL BSA). The cells were resuspended with 200 μL of binding buffer (DPBS containing 4.5 g/L glucose, 5 mM MgCl_2_, 1 mg/mL BSA, and 0.1 mg/mL tRNA) containing 250 nM of samples. After 50 min of incubation at 4°C, the samples were washed three times with washing buffer and analyzed by flow cytometry (Cytek Dxp Athena).

Uptake ability of DNA strands and conjugates measured by flow cytometry

Cells were seeded in a six-well plate at the density of 1 × 10^5^ per well and cultured for 24 h, followed by washing three times with DPBS. Next, 200 μL of culture medium containing 250 nM Cy5-XQ-2d, Cy5-S-XQ-2d, Cy5-MFSX, Cy5-MFC were separately incubated with cells for 2 h at 37°C and then removed. The cells were washed with DPBS, digested by trypsin, and resuspended by DPBS for flow cytometry (Cytek Dxp Athena).

Confocal imaging for targeted cellular uptake

PL45 cells were plated to optical dishes at a density of 1 × 10^5^ per dish and cultured for 24 h. Then they were washed with DPBS three times, followed by adding 200 μL of culture medium containing 250 nM Cy5-XQ-2d, Cy5-S-XQ-2d, Cy5-MFSX, Cy5-MFC separately and incubating for 2 h at 37°C. To explore the subcellular distribution, 75 nM LysoTracker Green DND-26 (Invitrogen) were added to the cells for 1 h, and the cells were imaged by ZEISS LSM880.

Stability of XQ-2d and S-XQ-2d *in vitro*

XQ-2d or S-XQ-2d (2 μM) in DMEM containing 20% FBS was incubated and shaken at 37°C for a certain time (0, 4, 8, 12, 24, 48, and 72 h), followed by heating to denature the nuclease at 95°C for 10 min. Then, all samples were analyzed by agarose gel electrophoresis. Generally, 10 μL of samples were mixed with 2 μL of 6× loading buffer, loaded into 12% native PAGE in running buffer (1× TBE, 9 mM Tris, 9 mM boric acid, and 1 mM EDTA, pH 8.0), and run for 40 min at 110 V. The gels were stained by Super Gelred^TM^ (10,000× in water, US Everbright^®^ Inc.) and imaged using the Bio-Rad ChemiDoc XRS system.

Endocytosis inhibition assay

PL45 cells were plated in a 12-well plate at a density of 1 × 10^5^ per well and cultured for 20 h. The cells were then preincubated with inhibitors at various concentrations for 45 min prior to the addition of 250 nM Cy5-XQ-2d or Cy5-S-XQ-2d. After 2 h, the cells were trypsinized and centrifuged (1000 r.p.m., 5 min), followed by removal of supernatants. After washing twice with PBS, the cells were resuspended in 200 μL of PBS. Fluorescence was determined by flow cytometry (Cytek Dxp Athena). Cells with DMSO were performed as a blank control to measure background signals which were subtracted from the final calculations.

Mouse models

Female BALB/c nude mice were obtained from Hunan SJA Laboratory Animal Co., Ltd. and used under protocols approved by Hunan University Laboratory Animal Center. Eight-week-old mice were devided into two groups and each group contained two mice. They were inoculated subcutaneously with 2 × 10^7^ PL45 cells in the left armpit. Tumors were observed 2 weeks after inoculation.

*In vivo* imaging and tissue distribution by biophotonic imaging analysis

Cy5-MFX and Cy5-MFSX at a single dose of 4.5 nmol were separately given to two groups of tumor-bearing nude mice via tail vein injection and imaged by the IVIS Lumina XR in vivo imaging system at a certain time (0, 0.5, 2, 4, 8, 12, 24, and 48 h). The mice were sacrificed 12 h and 48 h after injection. Tumors and major organs (hearts, lungs, livers, spleens, and kidneys) were collected for biophotonic imaging.

*Ex vivo* localization of Cy5-MFX and Cy5-MFSX in tumor sections

Tumor tissues were collected 48 h after injection, placed in 4% paraformaldehyde and stored at room temperature. The tissues were then incubated in 30% sucrose for 6 h, before embedding in optimal cutting temperature medium (OCT). The embedded tissues were then cryosectioned, washed, and permeabilized by 0.1% Triton X-100, followed by staining with DAPI, immunostaining, and analyzed by the digital slice scanning system (Pannoramic MIDI, 3DHISTECH) respectively.

Detection of plasma half-life and clearance

SD rats weighing 130 g each were injected with 100 μM 200 μL Cy5- MFX or Cy5-MFSX respectively. Fluorescence intensity in serum at different time points was measured, and concentration was calculated according to concentration-fluorescence intensity standard curves. The plasma clearance curve was presented by the relative fluorescence intensities of the conjugate over time, and the plasma half-life (*t*_1/2)_ was calculated by performing a logarithmic plot.

Cell cytotoxicity experiments

Cells were seeded at 5 × 10^3^ into a 96-well plate and incubated for 24 h, followed by adding fresh media containing a series of concentrations (0, 1.6, 8, 40, 200, and 1000 nM) of various drugs and incubating for 72 h. The supernatants were then discarded, and 100 μL of culture media containing 10% CCK-8 (New Cell & Molecular Biotech) were added to every well and incubated at 37°C for 1-2 h. The absorbance at 450 nm was measured by the Synergy 2 Multi-Mode microplate reader (Bio-Tek, Winooski, VT).

**Supplementary Figures**


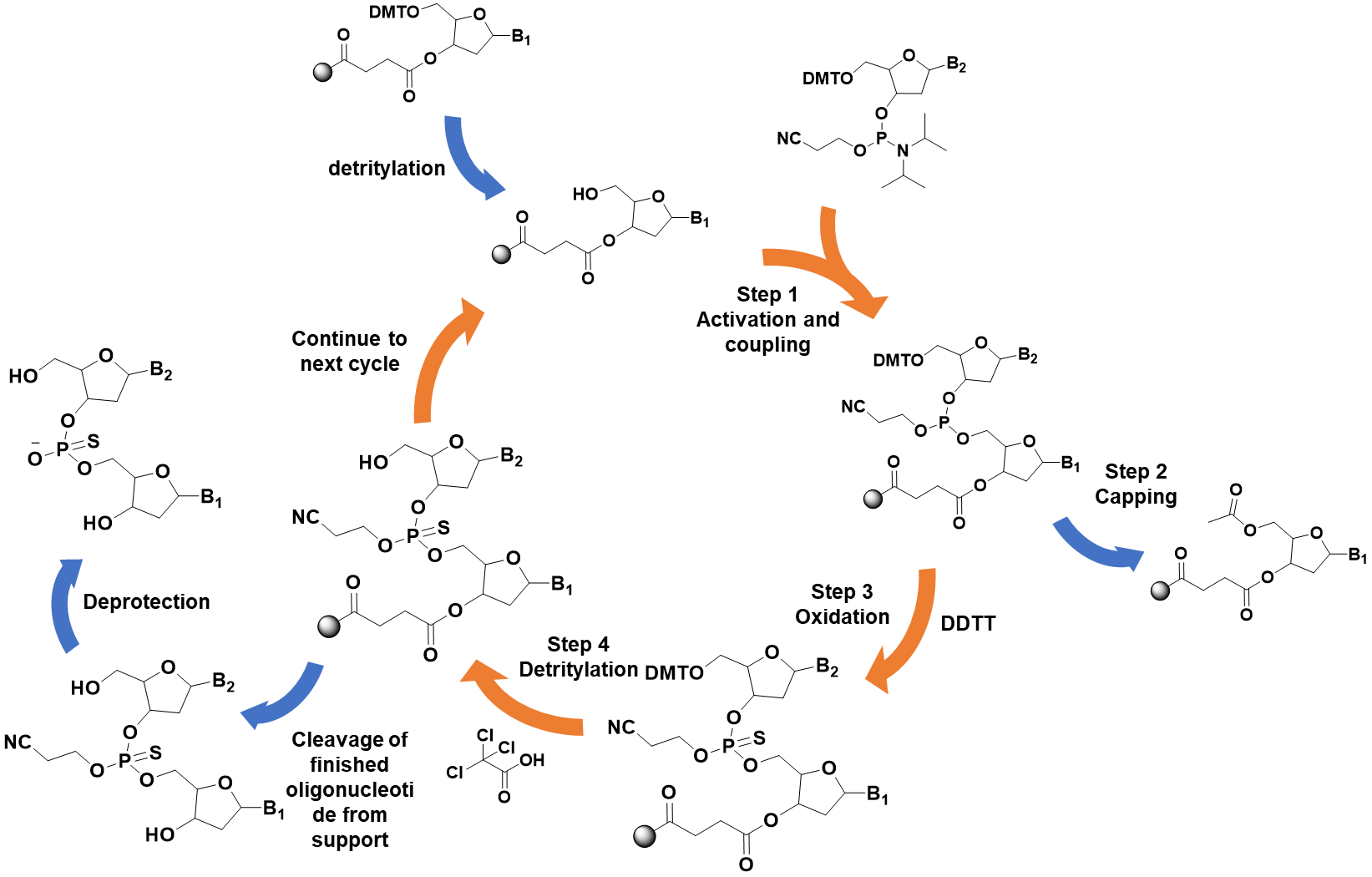


**Scheme S1** Synthetic route of phosphorothioate modified DNA strand.


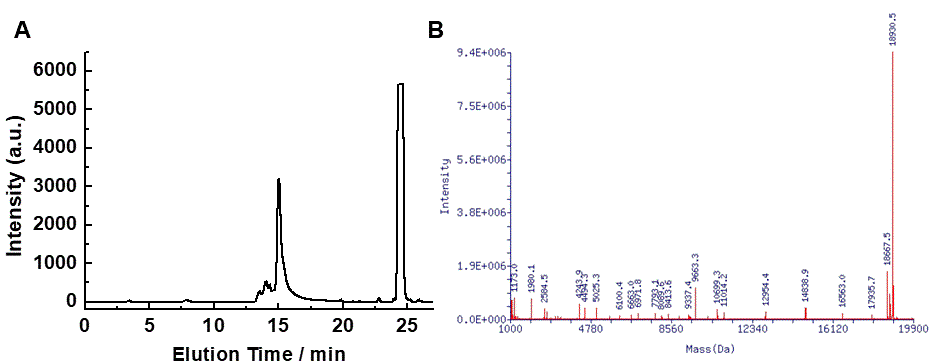


**Figure S1** Purification and Characterization of MFSX. A) HPLC trace of MFSX. B) Mass spectrum of MFSX.

**Figure S2** SPR sensorgram of the binding kinetics for the XQ-2d with immobilized CD71 (the black lines represent fitting the data).

**Figure S3** SPR sensorgram of the binding kinetics for the S-XQ-2d with immobilized CD71 (the black lines represent fitting the data).


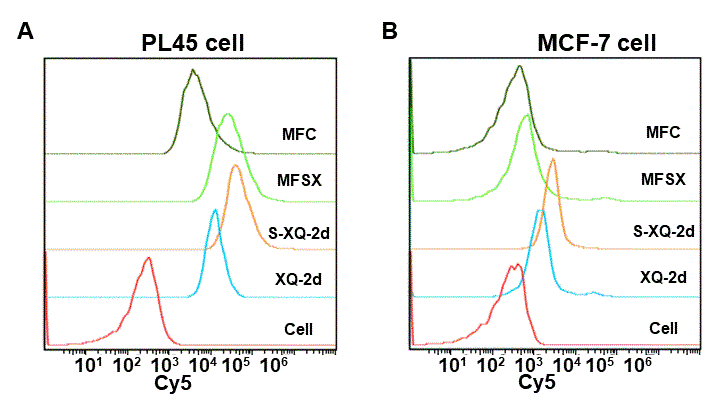


**Figure S4** Binding ability of 250 nM XQ-2d, S-XQ-2d, MFSX and MFC to A) PL45 cells and B) MCF-7 cells.


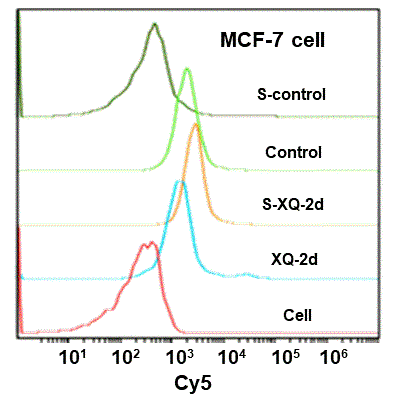


**Figure S5** Uptake amount of 250 nM Cy5-XQ-2d, Cy5-S-XQ-2d, Cy5-Control and Cy5-S-Control by MCF-7 cells incubated for 2 h at 37°C.


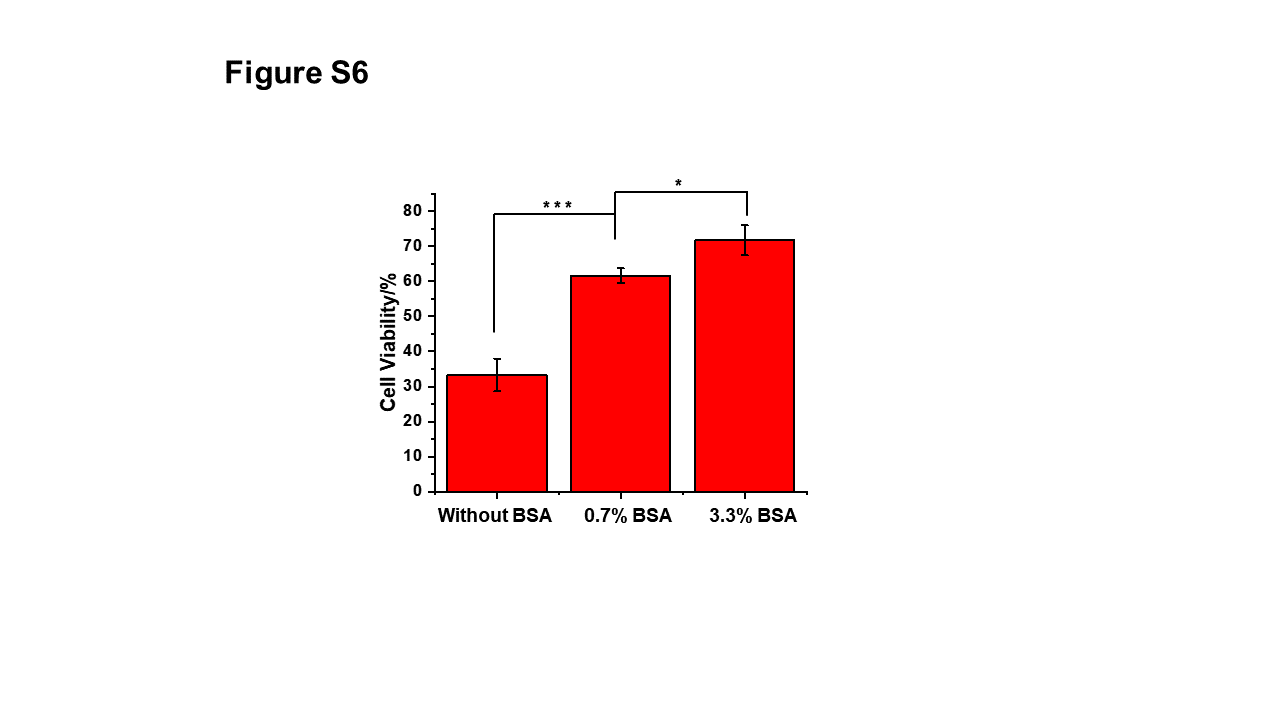


**Figure S6** Cell cytotoxicity of 1 μM MFSX with different amount of BSA for PL45 cell.


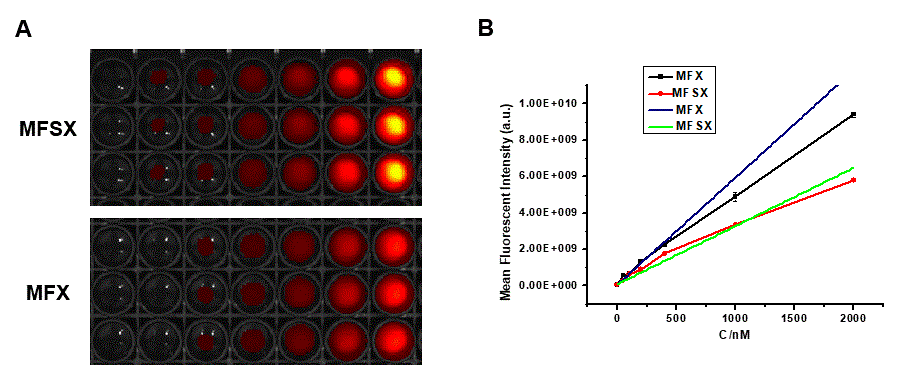


**Figure S7** Measurement and fit of standard curves for fluorescence intensity of Cy5-MFX or Cy5-MFSX in mouse blood. A) Imaging of Cy5-MFX or Cy5-MFSX in 100 μL mouse blood at the concentrations of 0, 0.05, 0.1, 0.2, 0.4, 0.8, and 1 μM (n=3). B) Calculation (with error bars) and fitted standard curves (without error bars) for concentration-dependent fluorescence intensity of MFX or MFSX.


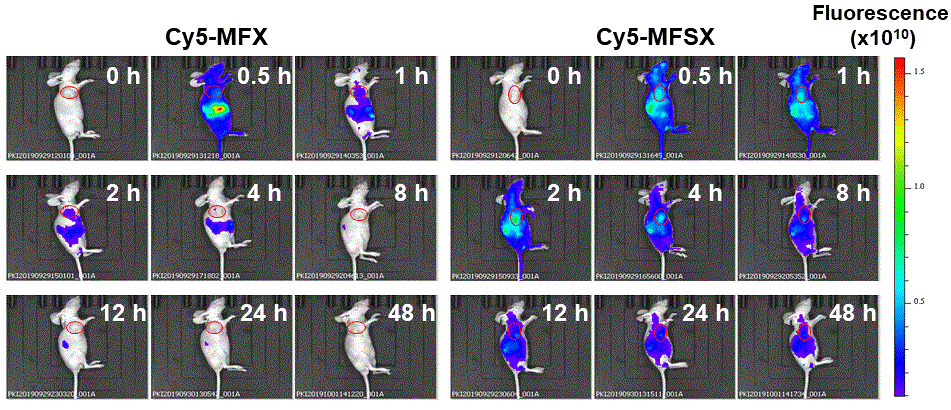


**Figure S8** *In vivo* imaging of 4.5 nmol Cy5-MFX or Cy5-MFSX at different times after injection.

**Table S1** Binding affinity of aptamers to CD71 Protein


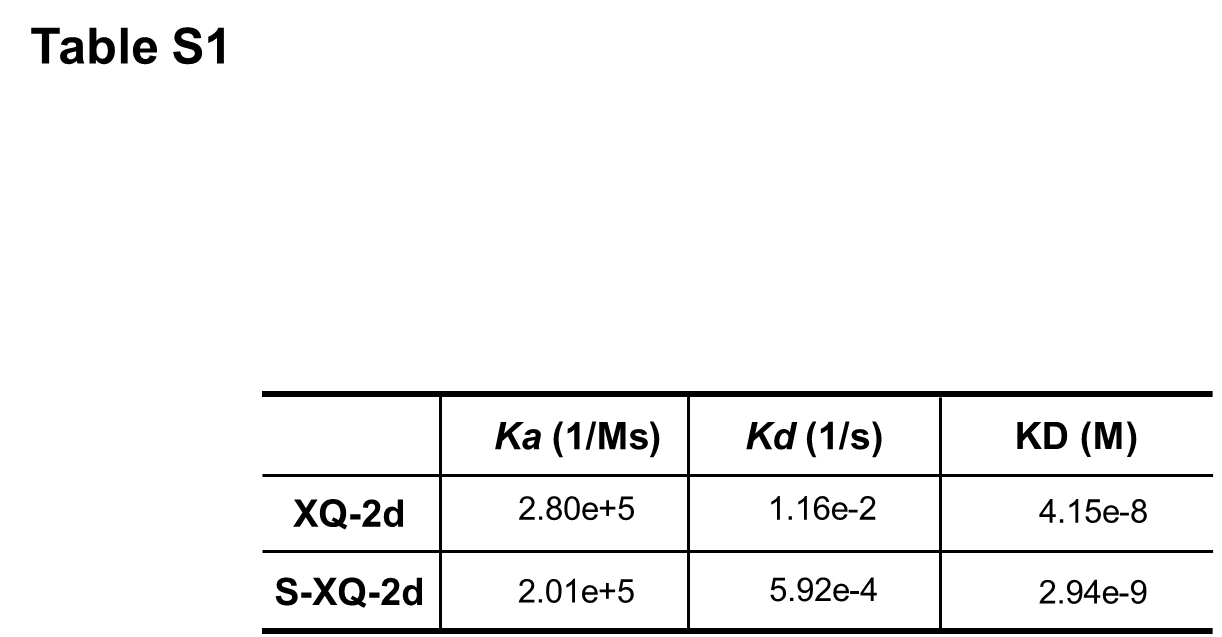


# The association rate constant (*Ka*), dissociation rate constant (*Kd*), and equilibrium dissociation constant (KD, KD = *Kd* / *Ka* ) were determined by SPR analysis.

**Table S2** The fitted half-life time and CL of MFSX and MFX


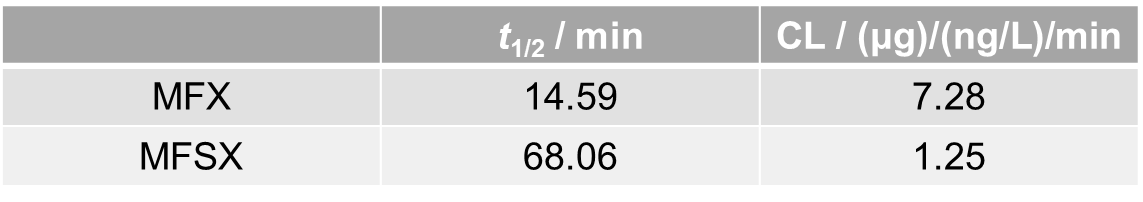

Supplement: nwad113_Supplemental_File [file nwad113_supplemental_file.docx]
